# Development and validation of a risk predication model for nutritional risk based on water swallowing test in post-stroke with dysphagia

**DOI:** 10.1371/journal.pone.0330982

**Published:** 2025-10-03

**Authors:** Ao Han, Shijiao Zhang, Ying Yao, Jingru He, Yingfang Wang, Huimin Yang

**Affiliations:** 1 School of Nursing, Henan University of Science and Technology, Luoyang, Henan province, China; 2 College of Basic Medicine and Forensic Medicine, Henan University of Science and Technology, Luoyang, Henan province, China; 3 Department of Respiratory and Critical Care Medicine, The First Affiliated Hospital of Henan University of Science and Technology, Luoyang, Henan province, China; 4 Emergency Department, The First Affiliated Hospital of Henan University of Science and Technology, Luoyang, Henan province, China; 5 Scientific research department, The First Affiliated Hospital of Henan University of Science and Technology, Luoyang, Henan province, China; Neighborhood Physical Therapy, UNITED STATES OF AMERICA

## Abstract

This study aims to develop and validate a prediction model for nutritional risk in Post-Stroke Dysphagia (PSD). This study retrospectively analyzed data on stroke patients with dysphagia from January 2022 to December 2023. A stepwise logistic regression model was used to construct the prediction model, and internal validation was performed using the bootstrap resampling (1000 iterations), the nomogram was developed for clinical applications. The final prediction model incorporated the following factors: age, marital status, mechanical ventilation, dysphagia treatment, fasting duration, atrial fibrillation, oral care frequency, serum potassium levels, and National Institute of Health stroke scale (NIHSS) score. The model demonstrated strong discriminatory power, with area under the ROC curve (AUC) values of 0.916 in the development set and 0.878 in the validation set. Calibration curves and the Hosmer-Lemeshow (H-L) test further confirmed the strong correlation between predicted and observed nutritional risks.The prediction model developed in this study exhibits high accuracy, consistency, and practical applicability, making it a valuable tool for predicting nutritional risk in PSD patients. The code library: https://osf.io/p3hjm.

## Introduction

Stroke is an acute neurological condition primarily resulting from disturbances in cerebral blood flow, often due to cerebrovascular diseases. It is classified into ischemic and hemorrhagic stroke [1]. According to the World Stroke Organization, over 12.2 million new stroke cases are reported globally each year, and approximately 6.5 million stroke-related deaths occur annually [2]. Stroke patients frequently experience various complications, with Post-Stroke Dysphagia (PSD) being one of the most common [3]. PSD is a functional disorder that impairs the safe and efficient transfer of food from the mouth to the stomach, with an incidence ranging from 22% to 70% [4]. The main causes of dysphagia are bulbar palsy resulting from bilateral cortical or brainstem tract injuries, and damage to the tongue and pharyngeal nerves, leading to symptoms such as excessive salivation, coughing, and ineffective swallowing.

Dysphagia not only increases the risk of complications like aspiration and lung infection, but also leads to reduced food intake and malnutrition in PSD patients due to neurological impairment and decreased daily activity [5,6]. Nutritional risk, distinct from malnutrition, refers to the potential impact of nutritional deficiencies on disease-related clinical outcomes, with higher incidence rates. This can result in poor functional recovery of hemiplegic limbs, more frequent infections, longer hospital stays, increased healthcare costs, reduced quality of life, and higher mortality [7,8].Therefore, nutritional risk screening is vital for improving PSD patient outcomes. The American Medical Review Committee recommends that hospitals complete nutritional screening within 24 hours of patient admission [9]. The American Association of Enteral and Parenteral Nutrition also emphasizes that nutritional risk screening is the first step in standardized nutrition management [10]. However, there are no uniform standards for screening tools. While commonly used tools like Nutritional Risk Screening 2002 (NRS-2002), Mini Nutritional Assessment-Short Form(MNA-SF), Malnutrition Universal Screening Tool (MUST), and Malnutrition Universal Screening Tool-Short Form (MNA-SF) are prevalent in clinics, their specificity is limited, and they are most effective for patients with clear awareness and good communication skills. These tools may not fully capture the nutritional risk factors of PSD patients, limiting screening accuracy and sensitivity.

Consequently, constructing a nutritional risk prediction model for PSD patients can enable early and accurate screening of high-risk groups, improving patient quality of life, reducing adverse health outcomes, and enhancing clinical management efficiency. This approach holds significant clinical value.

## Objectives

The objective of this study was to establish a nutritional risk screening nomogram for PSD patients based on the characteristics of PSD patients.

## Methods

### Study design and patients

We retrospectively collected the data of 601 eligible patients with PSD from the First Affiliated Hospital of Henan University of Science and Technology on April 30, 2024, with data from January 2022 to June 2023 used as the development set and data from 259 PSD patients between July 2023 and December 2023 as the validation set.

The inclusion criteria included: (1) age ≥ 18 years old; (2) stroke patients met the Diagnostic criteria of Diagnostic Points of Various Major cerebrovascular Diseases in China 2019 [11]; (3) water swallowing test (WST) ≥ grade Ⅲ. The exclusion criteria included: (1) previous history of dysphagia; (2) patients with hypermetabolic diseases such as tumors or hyperthyroidism; (3) have a history of mental illness; (4) same patient in different departments at the same time (retain complete information). The study was approved by The First Affiliated Hospital of Henan University of Science and Technology (No.2024-03-K0035).

### Diagnostic criteria

#### Water swallow test.

The Water Swallow Test (WST), first proposed by Toshio Koda in 1982 [12], is an assessment tool for rapid bedside evaluation of swallowing function. The patient is asked to sit and drink 30 ml of warm water at a normal speed. The grading system is as follows: Grade I: One attempt to drink, no choking. Grade II: No choking after drinking more than twice. Grade III: Coughing occurs after one attempt. Grade IV: Coughing occurs after two attempts. Grade V: Unable to drink all 30 ml, with obvious coughing. Grade I is considered normal; Grade II is suspicious; Grade III and above are abnormal.

#### Nutritional risk definition.

NRS-2002 [13] is an objective nutritional risk screening tool developed by the European Society of Parenteral and Enteral Nutrition (ESPEN) Expert Group in 2002. It has been proven effective and reliable for assessing patients’ nutritional status. The scale consists of three components: (1) impaired nutritional status score (up to 3 points); (2) the impact of disease severity on nutritional status (up to 3 points); (3) age score (1 point for age ≥ 70 years). The total score ranges from 0 to 7, with a score ≥3 indicating nutritional risk.

#### Sample size.

According to the Events Per Variable (EPV) principle [14], the number of outcome events should be at least 10 times the number of dependent variables to ensure the robustness of regression analysis. Based on domestic studies [15], the incidence of nutritional risk in PSD patients is 60.5%, so the required sample size is at least 607 cases. In total, 860 patients were included in the study.

#### Predictive variable selection.

Through literature reviews, group discussions, and consultations with neurology and nutrition experts, predictors of nutritional risk in PSD patients were identified, including: age, sex, medical insurance, marital status, residence, caregiver, WST, stroke type, National Institute of Health stroke scale (NIHSS), Activities of daily living (ADL) score, digestive history, hypertension, diabetes, atrial fibrillation, pulmonary infection, digestive complications, other complications, mechanical ventilation, use of ≥2 prescription drugs, nutritional support mode, dysphagia treatment, frequency of incidence, oral care frequency, surgery, C-reactive protein(CRP), body mass index (BMI), fasting duration, hospital stay, total protein level, hemoglobin, serum potassium, albumin, neutrophils, and serum sodium.

### Data collection

The research team consisted of two graduate students and one specialist nurse from the Department of Neurology. Data were collected through the hospital’s electronic medical record system based on the identified influencing factors. To ensure data accuracy and standardization, all investigators received uniform training before data collection, and two people checked whether the data was consistent.

### Data preprocessing

Cases with more than 20% missing data are excluded. For data with less than 20% missing data, multiple imputation was used to fill in the continuous variables; For missing categorical variables, the mode is used to fill in.

### Statistical analysis

Data analysis was performed using SPSS software (Version 25.0) and R software (Version 4.4.1). Continuous variables with a normal distribution are expressed as mean ± standard deviation (SD), and inter-group comparisons were made using the two-sample independent t-test. Non-normally distributed variables are presented as the median with interquartile range (IQR), and the Mann-Whitney U test was used for between-group comparisons. Categorical variables are presented as counts (%), and comparisons between groups were performed using the Chi-square test and Mann-Whitney U test. A *P* value < 0.05 was considered statistically significant.

First, univariate logistic regression was performed, and variables with a *P* value < 0.1 were included in multivariate logistic regression for independent correlation analysis. Multivariable regression was conducted using different methods, such as the inclusion method, forward selection, and backward elimination, creating models based on various variable sets. The optimal model was selected according to the minimum Akaike Information Criterion (AIC) [16], and a prediction model was developed.

**Fig 1:**
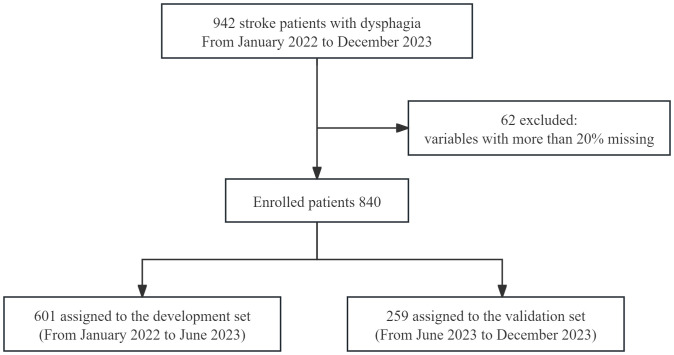
Shows a flowchart for building the prediction model development process.

The receiver operating characteristic (ROC) curve was used to estimate sensitivity and specificity, the larger the area under the ROC curve (AUC), the better the prediction performance of the model. The Hosmer-Lemeshow (H-L) test assessed the model, and the calibration curve was used to correct prediction accuracy. The decision curve analysis (DCA) evaluated the clinical applicability of the model. To assess model stability and prevent overfitting, bootstrapping was applied for internal validation. Fig 1 shows the flowchart of the prediction model development process. The code library: https://osf.io/p3hjm. Fig 1 Shows a flowchart for building the prediction model development process.

## Results

### Participant characteristics

A total of 942 patients were initially selected for the study. After excluding 82 patients with more than 20% missing data, 860 patients remained: 601 in the development set and 259 in the validation set. The overall incidence of nutritional risk was 64.30%, with 63.06% in the development set and 67.18% in the validation set. The basic characteristics and univariate analysis results of the development set are presented in Table 1.

**Table 1:** Demographic and clinical characteristics of the development set and validation set.

Variables	Total (n = 860)	Development set (n = 601)	Validation set (n = 259)	*p-*Value
Age, year	64.62 ± 13.14	64.72 ± 13.07	64.38 ± 13.34	0.727
Sex, n (%)				0.466
Male	522 (60.7)	360 (59.9)	162 (62.6)	
Female	338 (39.3)	241 (40.1)	97 (37.5)	
Medical insurance, n (%)				0.355
Worker with medical insurance	165 (19.20)	111 (18.5)	54 (20.9)	
Medical insurance for urban and rural residents	628 (73.0)	447 (74.4)	181 (69.9)	
Other	67 (7.8)	43 (7.2)	24 (9.3)	
Marital status, n (%)				0.600
Married	677 (78.7)	476 (79.2)	201 (77.6)	
Single/widowed/divorced	183 (21.3)	125 (20.8)	58 (22.4)	
Residence, n (%)				0.590
Cities and towns	327 (38.0)	225 (37.4)	102 (39.4)	
Countryside	533 (61.9)	376 (62.6)	157 (60.6)	
Caregiver, n (%)				0.235
Spouse	193 (22.4)	131 (21.8)	62 (23.9)	
Children	393 (45.7)	286 (47.6)	107 (41.3)	
Other	274 (31.9)	184 (30.6)	90 (34.8)	
WST, n (%)				0.224
Grade Ⅲ	523 (60.8)	359 (59.7)	164 (63.3)	
Grade Ⅳ	215 (25.0)	150 (25.0)	65 (25.1)	
Grade Ⅴ	122 (14.2)	92 (15.3)	30 (11.6)	
Stroke type, n (%)				0.811
Ischemic	475 (55.2)	333 (55.4)	142 (54.8)	
Hemorrhagic	366 (42.6)	256 (42.6)	110 (42.5)	
TIA	19 (2.2)	12 (2.0)	7 (2.7)	
NIHSS score, n (%)				0.385
0 ~ 1	10 (1.2)	7 (1.2)	3 (1.2)	
1 ~ 4	146 (17.0)	104 (17.3)	42 (16.2)	
5 ~ 15	212 (24.7)	154 (25.6)	58 (22.4)	
16 ~ 20	220 (25.9)	149 (24.8)	71 (27.4)	
21 ~ 42	272 (31.6)	187 (31.1)	85 (32.8)	
ADL, n (%)				0.737
Independent	16 (1.9)	14 (2.3)	2 (0.8)	
Mildly dependent	274 (31.9)	195 (32.5)	79 (30.5)	
Moderate dependent	387 (45.0)	259 (43.1)	128 (49.4)	
Heavy dependent	183 (21.3)	133 (22.1)	50 (19.3)	
Digestive history, n (%)				0.802
No	828 (96.3)	578 (96.2)	250 (96.5)	
Yes	32 (3.7)	23 (3.8)	9 (3.5)	
Hypertension, n (%)				0.657
No	305 (35.5)	216 (35.9)	89 (34.4)	
Yes	555 (64.5)	385 (64.1)	170 (65.6)	
Diabetes mellitus, n (%)				0.058
No	630 (73.3)	429 (71.4)	201 (77.6)	
Yes	230 (26.7)	172 (28.6)	58 (22.4)	
Atrial fibrillation, n (%)				0.572
No	790 (91.9)	550 (91.5)	240 (92.7)	
Yes	70 (8.1)	51 (8.5)	19 (7.3)	
Pulmonary infection, n (%)				0.535
No	379 (44.1)	269 (44.8)	110 (42.5)	
Yes	481 (55.9)	332 (55.2)	149 (57.5)	
Digestive complications, n (%)				0.057
No	581 (67.6)	418 (69.6)	163 (62.9)	
Yes	279 (32.4)	183 (30.4)	96 (37.1)	
Other complication, n (%)				0.690
No	633 (73.6)	440 (73.2)	193 (74.5)	
Yes	227 (26.4)	161 (26.8)	66 (25.5)	
Mechanical ventilation, n (%)				0.102
No	468 (54.4)	338 (56.2)	130 (50.2)	
Yes	392 (45.6)	263 (43.8)	129 (49.8)	
Use of ≥2 prescription drugs, n (%)				0.554
No	515 (59.9)	356 (59.2)	159 (61.4)	
Yes	345 (40.1)	245 (40.8)	100 (38.6)	
Nutritional support mode, n (%)				0.266
Autonomic feeding	238 (27.7)	178 (29.6)	60 (23.1)	
enteral nutrition	349 (40.6)	238 (39.6)	111 (42.9)	
parenteral nutrition	44 (5.1)	31 (5.2)	13 (5.0)	
Enteral and parenteral nutrition	229 (26.6)	154 (25.6)	75 (29.0)	
Dysphagia treatment, n (%)				0.178
No	805 (93.6)	567 (94.3)	238 (91.9)	
Yes	55 (6.4)	34 (5.7)	21 (8.1)	
Frequency of incidence, n (%)				0.837
First time	511 (59.4)	361 (60.1)	150 (57.9)	
Second time	284 (33.0)	195 (32.4)	89 (34.4)	
≥3 times	65 (7.6)	45 (7.5)	20 (7.7)	
Oral care frequency, times/day	2(0, 4)	2 (0, 4)	2 (0, 4)	0.185
Surgery, n (%)				0.847
No	552 (64.2)	387 (64.4)	165 (63.7)	
Yes	308 (35.8)	214 (35.6)	94 (36.3)	
CRP, n (%)				0.577
<6	198 (23.0)	138 (23.0)	60 (23.2)	
6-10	87 (10.1)	66 (11.0)	21 (8.1)	
>10	575 (66.9)	397 (66.0)	178 (68.7)	
BMI, n (%)				0.398
<18.5	25 (2.9)	14 (2.3)	11 (4.2)	
18.5 to 23.9	657 (76.4)	460 (76.5)	197 (76.1)	
24 to 27.9	136 (15.8)	99 (16.5)	37 (14.3)	
≥28	42 (4.9)	28 (4.7	14 (5.4)	
Fasting duration, day	0(0, 2)	0(0,2)	0 (0, 2)	0.507
Hospital time, day	13(7,24)	12(7,24)	13(7,23)	0.612
Total protein level, g/L	65.54 ± 7.96	65.38 ± 8.12	65.92 ± 7.57	0.365
Hemoglobin, g/L	133(120.75,146)	133(120,146)	134(121.41,146)	0.702
Serum potassium, mmol/L	3.78 ± 0.51	3.79 ± 0.50	3.76 ± 0.54	0.443
Albumin, g/L	39.5 (36.2, 42.6)	39.3 (35.9, 42.5)	39.7 (36.4, 43.2)	0.333
Neutrophil, 10^9/L	7.78 (5.19, 11.09)	7.72 (5.12, 11.16)	7.96 (5.24, 11.04)	0.557
Serum sodium, mmol/L	138.45(135.98,141)	138.20(135.94,140.8)	139(136,141.25)	0.294

### Risk factors for nutritional risk

In this study, nutritional risk was the dependent variable, and 34 risk factors were considered as independent variables. Univariate logistic regression in the development set identified 24 predictors of nutritional risk, including 4 demographic factors, 16 disease-related factors, and 4 laboratory indicators (Table 2). Risk factors with a P value < 0.1 in the univariate analysis were further analyzed using multivariate regression, and the final model inclusion was determined by the minimum AIC. The selected factors included age, marital status, NIHSS score, atrial fibrillation, mechanical ventilation, dysphagia treatment, oral care frequency, fasting duration, and serum potassium.

**Table 2:** Univariate and multivariate logistic regression analysis were used to analyze the risk factors of nutritional risk in PSD.

Variables	Univariable		Multivariable	
	OR (95%CI)	*p*-Value	OR (95%CI)	*p*-Value
Age	1.05(1.04-1.07)	<0.001	1.1(1.07-1.13)	<0.001
Sex	1.09(0.78-1.54)	0.602		
Medical insurance				
Urban and rural residents	1.9(1.25-2.9)	0.003	1.17(0.61-2.25)	0.641
Other	0.72(0.36-1.47)	0.37	0.43(0.15-1.26)	0.124
Residence	0.85(0.61-1.21)	0.372		
Marital status	3.62(2.19-5.99)	<0.001	2.1(1.04-4.23)	0.039
Caregiver				
Children	1.41(0.93-2.14)	0.104		
Other	5.54(3.3-9.31)	<0.001		
WST				
Grade 4	1.24(0.83 ~ 1.86)	0.291		
Grade 5	0.86(0.54 ~ 1.37)	0.515		
Stroke type				
Hemorrhagic	2.32(1.63-3.3)	<0.001		
TIA	4.2(0.91-19.44)	0.067		
NIHSS score				
1 ~ 4	2.21(0.25-19.2)	0.472	2.78(0.19-40.95)	0.457
5 ~ 15	4.15(0.49-35.32)	0.192	3.5(0.24-50.19)	0.356
16 ~ 20	62.77(7.02-561.57)	<0.001	27.3(1.78-419.46)	0.018
21 ~ 42	25.17(2.94-215.66)	0.003	15.67(1.08-227.9)	0.044
ADL				
Mildly dependent	7.61(0.98-59.35)	0.053		
Moderate dependent	31.89(4.1-248.27)	0.001		
Heavy dependent	144.18(17.23-1206.79)	<0.001		
Digestive history	1.35(0.55-3.34)	0.511		
Hypertension	0.95(0.67-1.34)	0.753		
Diabetes mellitus	0.75(0.52-1.07)	0.114		
Atrial fibrillation	10.59(3.26-34.38)	<0.001	6.51(1.61-26.38)	0.009
Pulmonary infection	4.86(3.4-6.95)	<0.001		
Digestive complications	1.61(1.11-2.33)	0.013		
Other complication	2.13(1.43-3.2)	<0.001		
Mechanical ventilation	9.27(6.04-14.21)	<0.001	2.69(1.35-5.35)	0.005
Use of ≥2 prescription drugs	0.71(0.51-1)	0.048		
Nutritional support				
Enteral nutrition	6.22(3.81-10.16)	<0.001		
Parenteral nutrition	3.91(2.59-5.9)	<0.001		
Enteral and parenteral	8.82(3.23-24.07)	<0.001		
Dysphagia treatment	0.3(0.14-0.61)	0.001	0.37(0.13-1.02)	0.055
Frequency of incidence				
Second time	0.76(0.41-1.43)	0.395		
≥3 times	0.91(0.63-1.3)	0.605		
Oral care frequency	2.02(1.79-2.28)	<0.001	1.41(1.13-1.75)	0.002
Surgery	2.35(1.63-3.41)	<0.001		
CRP				
6-10	0.57(0.37-0.87)	0.009		
>10	0.62(0.33-1.16)	0.136		
BMI				
18.5-23.9	0.16(0.03-0.77)	0.022		
24-27.9	0.11(0.02-0.58)	0.009		
≥28	0.33(0.07-1.51)	0.155		
Fasting duration	2.03(1.69-2.43)	<0.001	1.45(1.14-1.85)	0.003
Hospital time	1.01(1-1.02)	0.245		
Total protein level	1.01(0.99-1.03)	0.582		
Hemoglobin	0.99(0.98-1)	0.038		
Serum potassium	0.56(0.4-0.79)	0.001	0.37(0.22-0.63)	<0.001
Albumin	0.96(0.93-0.99)	0.008		
Neutrophil	1.09(1.04-1.13)	<0.001		
Serum sodium	0.99(0.96-1.02)	0.565		

### Model development

**Fig 2:**
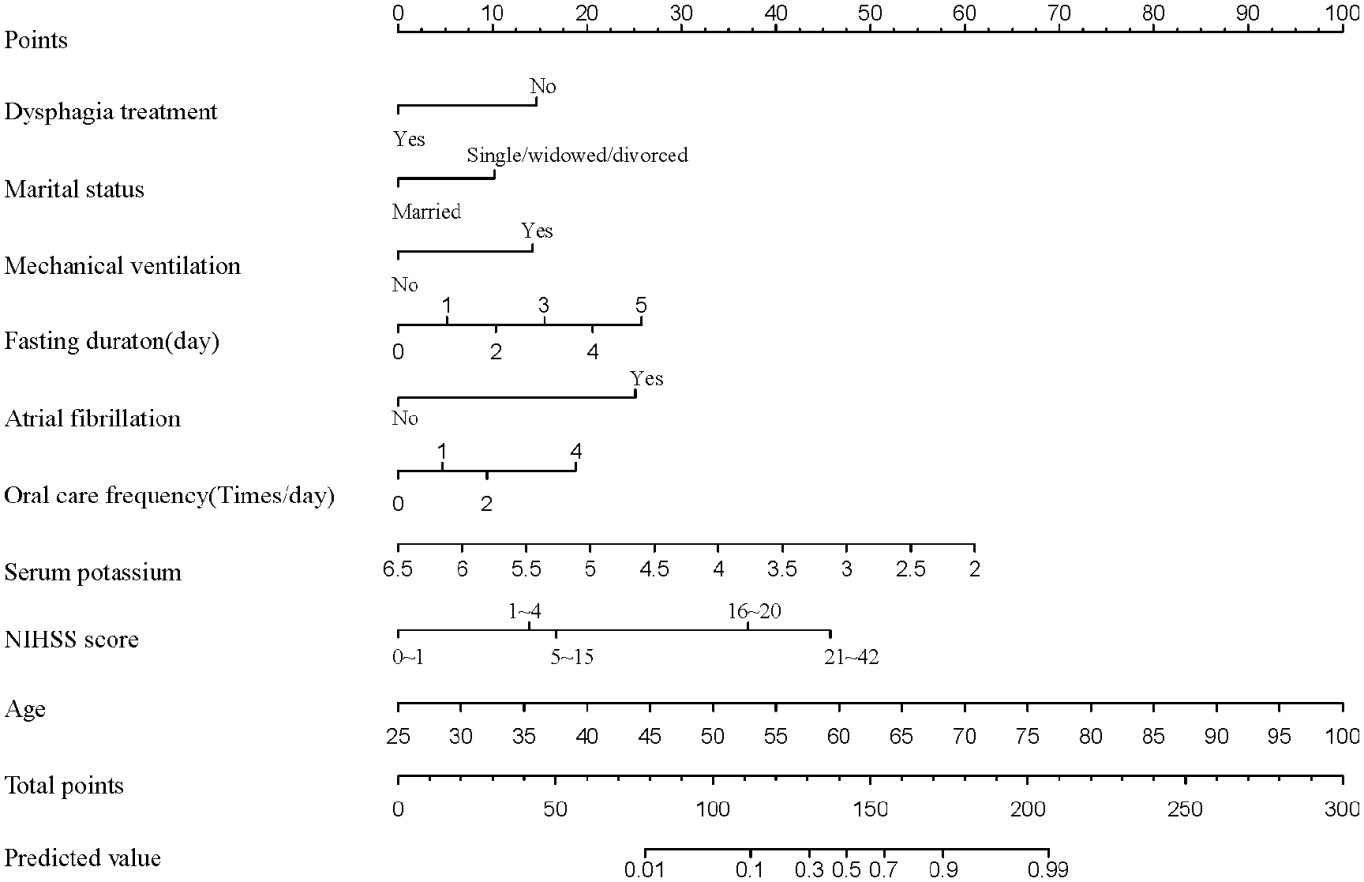
Nomogram prediction of nutritional risk in PSD. As a predictive tool for PSD nutritional risk, nomogram assigns each factor a score based on a scoring system. The sum of these points produces an overall score that, when aligned with the scale, provides the likelihood that PSD nutritional risk will occur.

Based on multivariate analysis results, a nomogram model was developed using nine independent factors to predict nutritional risk in PSD patients (Fig 2). How to use Nomogram: Within 24 hours of the patient’s admission, the medical staff scored each predictor according to the actual situation of the patient, added the total score, and projected it into the Predicted value, which is the predicted probability of nutritional risk, and the clinical staff were divided into two groups: high risk and low risk according to the optimal cut-off value of 0.672. For example, a person aged 60 years (47.5 points), no swallowing function therapy (15 points), divorced (10 points), no mechanical ventilation (0 points), fasted for 1 day (5 points), had a history of atrial fibrillation (25 points), had 1 oral care per day (5 points), blood potassium concentration was 3.5 (41 points), NIHSS score was between 5 ~ 15 points (17.5 points), and the total score was 166, and the corresponding probability of nutritional risk may occur is 82%. The optimal cut-off values of this model were 0.672, 0.82 > 0.672, suggesting that the patient was a high-risk group and should be intervened as soon as possible. Fig 2. Nomogram prediction of nutritional risk in PSD.

### Internal and external model validation

**Fig 3:**
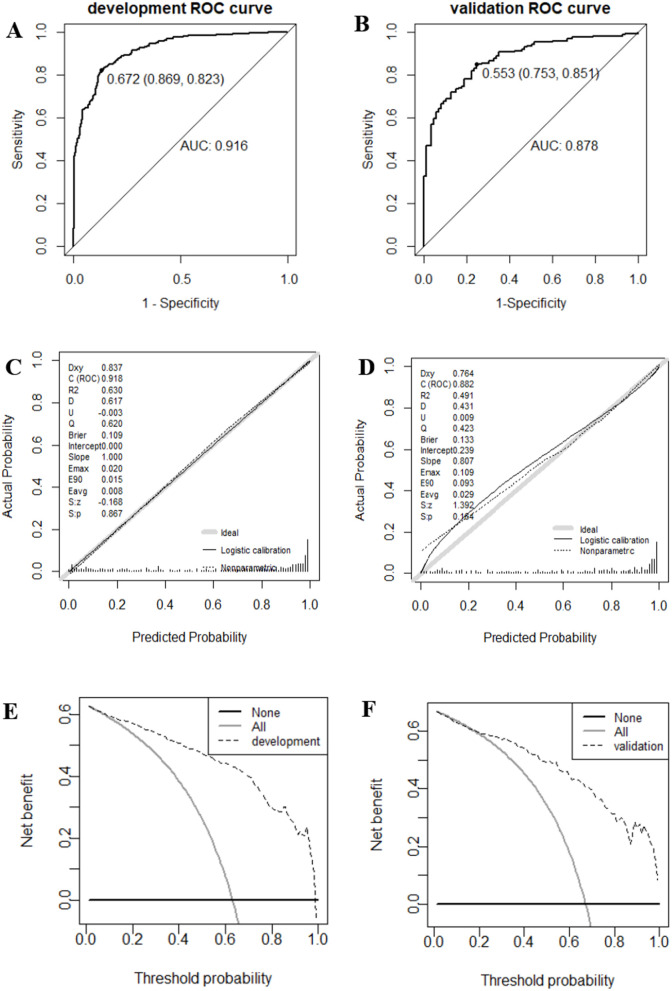
Performance evaluation of the PSD nutritional risk prediction model.

R4.4.1 was used to generate the ROC curve of the prediction model. The AUC of the development set was 0.916 (95% CI:0.893–0.938) (Fig 3A). After 1000 bootstrap iterations for internal verification, the mean AUC of the model was 0.921 (95% CI: 0.893–0.916), indicating that the model has strong discriminant ability. The accuracy and specificity of the model in the developmental concentration were 0.842 and 0.881, respectively. The calibration plot (Fig 3C) and the H-L test (χ² = 9.979, **P* *= 0.267) supported the prediction accuracy of the model. The DCA (Fig 3E) shows that this model provides a greater net benefit than a “treat all” or “treat none” strategy. When the optimal critical value of the model is 0.672 calculated based on the Youden index, the sensitivity is 0.869 with a specificity of 0.823. Fig 3. Performance evaluation of the PSD nutritional risk prediction model.

For external validation, 259 PSD patients observed between June and December 2023 were included. The model’s AUC was 0.878 (95% CI: 0.837–0.920) (Fig 3B). The H-L test result was χ² = 8.666, **P* *= 0.371, and the accuracy and specificity were 0.819 and 0.891, respectively, confirming the model’s strong predictive power in the external validation set. Calibration curves (Fig 3D) and DCA (Fig 3F) further support the model’s discriminatory ability and clinical utility.

## Discussion

In this study, 601 PSD patients were included in the development set, with a nutritional risk incidence of 63.06%, slightly higher than the 58.9% reported in the Yoon study [17]. This discrepancy may be attributed to differences in geographical factors and screening tools. The nutritional risk nomogram prediction model developed in this study is characterized by its simplicity, rapid operation, and strong predictive performance. The AUC values were 0.916 for the development set and 0.878 for the external validation set. The H-L test showed a *P* value greater than 0.05, indicating good model fit. Compared with the studies of Gao [18], Lin [19], Duan [20], Liu [21] and Zeng [22], the AUC value of this study was higher than that of previous studies (0.819, 0.800, 0.871, 0.860, 0.854), and the ability of the model to distinguish whether PSD patients had nutritional risk was significantly improved. The overall performance of a prediction model is affected by the choice of predictor variables, the size of the sample size, the handling of missing and outlier values, and the method used to construct the model [23]. The source of the predictor variables was not clearly indicated in previous studies, but this study was based on the results of literature review and expert consultation, and combined with the actual clinical situation, the predictors were finally included in the model construction, so that the scientific and clinical practicability of the model were guaranteed. In addition, the small sample size and low quality of the included data can lead to model overfitting and affect the accuracy of the prediction results [24]. Lin [19]included only 92 patients, while Liu [21] included the largest sample size of 325 in the existing study. In this study, the sample size was further expanded, 601 patients with PSD were included for model construction, and < 20% of the missing data were multi-imputed, which can increase the reliability and stability of the model to a certain extent. These results demonstrate that the model possesses strong discriminative ability and clinical utility in identifying nutritional risk, making it effective for preliminary screening of high-risk patients.

This study identified several factors influencing nutritional risk in PSD patients, including age, marital status, use of mechanical ventilation, dysphagia treatment, fasting duration, history of atrial fibrillation, oral care frequency, serum potassium levels and NIHSS score. The included predictive factors are all objective assessment indicators, which avoid the information bias introduced by traditional nutritional assessment tools that rely on patients’ recollections.

This study found that the risk of nutritional risk in PSD patients increases with age. Previous research has shown that older individuals tend to experience poorer recovery of various functions, including gastrointestinal decline, poor oral hygiene, reduced ability to eat independently, and increased susceptibility to gastrointestinal complications and hemiplegic limb dysfunction [17,25]. These factors contribute to a higher incidence of nutritional risk in the elderly. Additionally, this study revealed that PSD patients without spouses were approximately 3.62 times more likely to face nutritional risks than those with spouses, consistent with previous findings [26,27]. Spouses play a crucial role in providing emotional support, assisting with daily activities, and boosting recovery confidence, which can help reduce the incidence of nutritional risk.

The study found that PSD patients with a high NIHSS score are more prone to nutritional risk. Research indicates that individuals with severe neurological impairment often experience reduced daily functioning, difficulty swallowing, facial or limb weakness, and cognitive deficits, all of which significantly impair eating behaviors and food intake, leading to inadequate nutrition. Moreover, PSD patients are at a higher risk of coughing and aspiration during eating, which can result in complications such as lung infections, further increasing the incidence of nutritional risk. Numerous studies have demonstrated that dysphagia is closely associated with the nutritional status of patients [28–30]. In particular, PSD patients are more prone to aspiration, which can lead to complications like lung infections. dysphagia treatment, such as sensory neuromuscular electrical stimulation [31], cold stimulation [32], acid stimulation [33], and acupuncture [34], have been shown to help restore swallowing ability, improving nutritional status, reducing complications, shortening hospital stays, and ultimately enhancing patient prognosis. Additionally, PSD patients with a history of atrial fibrillation are at greater risk of nutritional imbalances. Atrial fibrillation increases the likelihood of severe stroke, and more severe strokes often result in greater nutritional deficits. This can lead to prolonged stays in the ultra-acute stroke unit and an increased risk of early infections, disability, and mortality [35].

Hemorrhagic stroke patients often require fasting in the early stages due to issues like intestinal edema, ulcers, and dysbiosis, with parenteral nutrition provided during this period. However, this can lead to complications such as hyperglycemia and infection [36]. Studies have also shown that patients receiving parenteral nutrition are more likely to require subsequent mechanical ventilation [37]. ventilation In this study, 63.71% of fasting patients underwent mechanical ventilation, which further increased the likelihood of nutritional risk [38–40]. Mechanical ventilation contributes to elevated intra-abdominal pressure, leading to mucosal damage, digestive tract bleeding, and digestive dysfunction, all of which impair nutrient absorption and increase nutritional risks. Additionally, the study found that more frequent daily oral care was associated with a higher nutritional risk, which aligns with previous research [41]. On the one hand, stroke patients with a small number of teeth, poor oral health, and weak daily activities often need frequent oral care, and such patients have more severe disease and are more prone to nutritional risks in hypermetabolic conditions [42]. On the other hand, Dysphagia can result in a loss of self-care ability and poor oral hygiene, which in turn affects appetite and food intake, leading to nutritional deficiencies and an increased risk of nutritional problems [43]. Lastly, lower serum potassium levels were found to correlate with a higher risk of nutritional issues. Stroke patients often experience potassium metabolism disorders, which place additional strain on the nervous system. Coupled with dysphagia and insufficient nutrition intake, this prolongs rehabilitation and increases the risk of stroke recurrence and nutritional complications [44].

## Conclusions

The condition of PSD patients is complex and dynamic, with a high incidence of nutritional risk. This risk is closely associated with various factors, including age, marital status, mechanical ventilation, dysphagia treatment, fasting duration, atrial fibrillation, oral care frequency, serum potassium and NIHSS score. By constructing a prediction model based on the WST score, healthcare professionals can accurately screen for nutritional risk in PSD patients. This model is crucial for developing personalized nutrition interventions, improving patient nutritional status, enhancing quality of life, and ultimately improving clinical outcomes in PSD patients.
